# Effect of GLP-1/GLP-1R on the Polarization of Macrophages in the Occurrence and Development of Atherosclerosis

**DOI:** 10.1155/2021/5568159

**Published:** 2021-03-27

**Authors:** Li Yang, Long Chen, Dongfeng Li, Hao Xu, Jishun Chen, Xinwen Min, Meian He, Tangchun Wu, Jixin Zhong, Handong Yang, Jun Chen

**Affiliations:** ^1^Affiliated Dongfeng Hospital, Hubei University of Medicine, Shiyan, China; ^2^School of Public Health, Tongji Medical College, Huazhong University of Science and Technology, Wuhan, China; ^3^Department of Rheumatology and Immunology, Tongji Hospital, Huazhong University of Science and Technology, Wuhan, China

## Abstract

**Aims:**

To investigate the effect of GLP-1/GLP-1R on the polarization of macrophages in the occurrence and development of atherosclerosis.

**Methods:**

Totally, 49 patients with coronary heart disease (CHD) and 52 cases of health control (HC) were recruited, all subjects accept coronary angiography gold standard inspection. One or more major coronary arteries (LM, LAD, LCx, and RCA) stenosis degree in 50% of patients as CHD group; the rest of the stenosis less than 50% or not seen obvious stenosis are assigned to the HC group. Flow cytometry were used to detect the percentage of (CD14+) M macrophages, (CD14+CD80+) M1 macrophages, (CD14+CD206+) M2 macrophages, and their surface GLP-1R expression differences in the two groups, using BD cytokine kit to detect the levels of IL-8, IL-1*β*, IL-6, IL-10, TNF, and IL-12p70.

**Results:**

GLP-1R expression on the surface of total macrophages and M2 macrophages was different between the CHD group and the HC group (*P* < 0.05). There was no difference in the percentage of total, M1 or M2 macrophages (*P* > 0.05). Concentration of IL-8 in the HC group was higher than that in the CHD group (*P* < 0.05). There is no significant difference in the cytokine IL-1*β*, IL-6, IL-10, TNF, and IL-12p70 in the two groups (*P* > 0.05). After controlling for potential confounders including age, gender, smoking status (S.S.), drinking status (D.S.), HR, SBP, DBP, PP, TC, TG, HDL-C, LDL-C, GHbA1c, M, M1, M2, GLP-1R_M, GLP-1R_M1, GLP-1R_M2, IL-8, IL-1*β*, IL-6, IL-10, TNF, and IL-12p70 by multiple linear regression, decreasing Gensini Score was significantly associated with increased percentage of M1 macrophage.

**Conclusion:**

GLP-1R agonist is independent of the hypoglycemic effect of T2DM and has protective effect on cardiovascular system. GLP-1R may regulate the polarization of macrophages toward M2, thus playing a protective role in the progression of coronary atherosclerosis.

## 1. Introduction

Type 2 diabetes mellitus (T2DM) is a major risk factor for coronary atherosclerotic heart disease (CHD); CHD is a severe and fatal complication of T2DM [1]. Patients with long course of disease more than 10 years often have retinopathy, which is one of the main causes of blindness and seriously affects the quality of life of the patients [2]. More importantly, atherosclerosis (AS) is the pathological basis of malignant cardiovascular and cerebrovascular events (MACE) such as CHD, acute myocardial infarction (AMI), and stroke [3]. Although AS has always been the core of the research field in cardiovascular and cerebrovascular diseases, this complex disease has still not been conquered by human beings.

Studies have found that a variety of chronic noncommunicable diseases, such as essential hypertension (EH), T2DM, AS, obesity, and nonalcoholic fatty liver disease (NAFLD), are not only related to genetic, environment, and metabolic disorders [4] but also a mild systemic chronic inflammatory reaction; both inflammation and immune response are involved in its occurrence and development to some extent [5]. Macrophages are innate immune cells, which not merely damage vascular endothelial cells as an initial factor in the process of atherosclerosis, more than run through the whole process of the development of atherosclerosis [6]. Foam cells formed after the phagocytosis of ox-LDL and further release inflammatory cytokines, which also play a vital role in AS [6]. In addition, adaptive immunity represented by T cell subsets such as Th (helper T cell) and Treg (regulatory T cell) is also involved, and other Th2, Th9, Th17, and Th22 subsets remain to be studied [7, 8].

Glucagon-like peptid-1 receptor (GLP-1R) is a G protein-coupled receptor [9]; animal studies shows that activation of GLP-1/GLP-1R pathway can benefit mice with AS, and the mechanism involves the polarization of immune cells [10]. However, most of the current studies have been done at the cellular or animal level. The distribution of GLP-1R on human immune cells and whether it mediates the polarization of human macrophages and then independently make an antiatherosclerosis impact in patients without T2DM is unclear. Therefore, this paper intends to use flow cytometry to analyze the distribution of M1 (proinflammatory) and M2 (anti-inflammatory) macrophages as well as their surface GLP-1R in patients with different degrees of CHD and preliminarily explore the role of cytokine network in GLP-1R-mediated immune cell polarization.

## 2. Methods

### 2.1. Subjects

All participants with proposed diagnosis of coronary atherosclerosis (CHD) who were admitted to the Department of Cardiology, Dongfeng General Hospital of affiliated to Hubei University of Medicine, from August to September 2020, people who had clinical symptoms such as chest tightness, were collected after the informed consent. All subjects accept gold standard of cardiac angiography inspection, one or more major coronary arteries, left main (LM), left anterior descending (LAD), left circumflex (LCx), and right coronary artery (RCA) stenosis degree in 50% of patients as CHD group, the rest of the stenosis less than 50%, or no obvious stenosis are assigned to the health control (HC) group. Inclusion criteria are as follows: the Han nationality, chronic myocardial ischemia syndrome, and acute coronary syndrome; all underwent cardiac angiography. Exclusion criteria are as follows: (1) secondary or gestational diabetes mellitus; (2) taking hormonal drugs, such as thyroid hormone or glucocorticoids; (3) rheumatoid arthritis and other autoimmune diseases; (4) severe cardiac, liver, and renal insufficiency; (5) malignant tumor; (6) acute diseases other than myocardial infarction, such as aortic dissecting aneurysm. Finally, 101 people who met the requirements were included in the analysis.

### 2.2. Data Collection and Experimental Methods

General information was collected for all subjects, including age, sex, smoking, alcohol consumption, heart rate (HR), systolic and diastolic blood pressure (SBP, DBP), and differential pulse pressure (PP). Gensini score was completed according to the guide in 2009 [11]. The peripheral venous blood of fasting patients was numbered in time, and triglyceride (TG), total cholesterol (TC), low-density lipoprotein cholesterol (LDL-C), high-density lipoprotein cholesterol (HDL-C), and very low-density lipoprotein cholesterol (VLDL-C) were detected by enzymatic method using automatic biochemical analyzer. Determination of glycosylated hemoglobin A1c (GHbAc1) by High Pressure Liquid Chromatography (HPLC).

### 2.3. Detection of GLP-1R Expression of Macrophages

Blood samples were stored 4°C after sampling and tested within 8 hours to ensure the number of living cells in each sample. Clusters of immune cells are as follows: 1 *μ*L CD3+-labeled antibody, 5 *μ*L CD4+-labeled antibody, 5 *μ*L CD80+-labeled antibody, 10 *μ*L CD14+-labeled antibody, 10 *μ*L CD80+-labeled antibody, and 10-*μ*L CD206+ labeled antibody were added to a 2 mL EP tube and mixed with vortex 3 s. 10 *μ*L antibody was added to the absolute count microsphere test tube (No. Z6410004-10, microsphere number: 51300). Reverse absorption of 30 *μ*L EDTA-K2 anticoagulant whole blood from suspected CHD patients was added to the bottom of the tube. After mixed with vortex 3 s, the blood was incubated at room temperature and dark for 25 min. The hemolytic agent 460 *μ*L for flow cytometer diluted to 1 time was added into the tube, and the tube cap was placed on the tube and gently vortexed for 15 s. The solution was kept away from light for 10 min at room temperature.

Flow cytometer setting is as follows: NovoExpress software was used to conduct automatic sampling cell analysis. The six channel parameters were B530 (CD14 FITC), B572 (CD206 PE), B675 (CD80 PE-CY7), R675 (GLP-1R APC), and R780 (CD4 APC-CY7). Stopping conditions: 5000 M1 macrophages or sample volume up to 450 *μ*L flow rate: high speed, 66 *μ*L/min; sample flow diameter: 16.8 *μ*m well. Plate management: mixing once every 3 wells, flushing once every 3 wells. Blending parameter: speed: 1500 rpm, acceleration time: 2 s, duration: 10 s. Flow cytometry was completed by two experienced personnel and conducted in a blind method with unknown grouping of each sample, so as to avoid systematic errors caused by exposure suspicion bias caused by subjective factors.

### 2.4. Detection of Cytokines

The collected whole blood anticoagulant tube was put into a centrifuge, centrifuged at 500 g for 5 min, and the upper plasma was absorbed and stored in the cryopreserved tube, which was stored in the -80°C refrigerator for later use. The BD cytokine kit (No. 551811) was used for quantitative analysis of IL-8, IL-1*β*, IL-6, IL-10, TNF, and IL-12p70.

Preparation of inflammatory cytokine standard: draw 2 mL determination of dilution in a bottle of freeze-dried inflammation factor standard in the bottle, with liquid moving head gently mix recombinant protein, balance for 20 min at room temperature, then transfer the standard to 15 mL polypropylene tube, marked for the highest concentration of standard (5000 pg/mL), other eight 15 mL polypropylene pipe marked as 1 : 2, 1 : 4, 1 : 8, 1 : 16, 1 : 32, 1 : 64, and 1 : 128, respectively, one more negative control was also labeled.

Mixed human inflammatory cytokines capture microspheres: six bottles of capture microspheres were mixed, each time before complete vortex capture microspheres 3-5 s to make it completely suspended. The above mixed capture microspheres were centrifuged at 200 g for 5 min, carefully absorbed and discarded the supernatant 30 *μ*L, added the equal volume of serum enhanced buffer suspension, in order to reduce the false-positive rate of plasma type samples containing protein, and incubated at room temperature in dark for 30 min.

Plasma sample detection procedures: all eligible plasma samples were stored at -80°C, immediately placed on the ice to thaw. Vortex to mix all plasma samples in the sterile tube, centrifuge 500 g for 5 min. Add 30 *μ*L mixed capture beads to all EP tubes, and vortex the capture beads every 5 EP tubes. Number each EP tube; add 30 *μ*L each sample to the numbered EP tube and incubated in dark for 1.5 hours at room temperature. 1 mL washing buffer was added to each EP tube then centrifuge at 200 g for 5 min. Carefully and continuously absorb and discard the supernatant, leaving about 100 *μ*L of liquid in each EP tube. Add 30 *μ*L human inflammatory cytokine PE secondary antibody to all EP tubes and stir gently. Hatch the EP tube at room temperature for 1 hour without light. Add 1 mL washing buffer to each tube and centrifuge at 200 g for 5 min again. Carefully extract and discard the supernatant from each EP tube. 300 *μ*L washing buffer was added to each EP tube to resuspend the captured microspheres. Samples are transferred to 96-well plates for testing, and random injection sequence was adopted for cytokine detection of all plasma samples.

The cells were automatically collected and analyzed using NovoExpress software. The six-channel parameters were B530 (FITC), B572 (PE), B67 (Per-CP), B780 (PE-CY7), R675 (APC), and R780 (APC-CY7). Stopping conditions: 1800 captured microspheres or samples of 100 *μ*L volume were detected. Flow rate: low speed, 14 *μ*L/min. Flow diameter: 7.7 *μ*m. Threshold: FSC-H greater than 10000, SSC-H greater than 5000 well. Board management: mixing once per well, flushing once per well. Mixing parameters: speed 1000 rpm, acceleration time 0 s, and duration 5 s.

FCAP Software parameters: scattering parameters: FSC-A, scattering peak: 5, clustering parameters: APC-A, reporting parameters: PE-H microsphere A1, A2, A3, A4, A5, and A6 corresponding IL-8, IL-1*β*, IL-6, IL-10, TNF, and IL-12p70. Quantitative analysis of mixed human cytokine concentrations of standard curve theory in order: 0.00 pg/mL, 19.53 pg/mL, 39.06 pg/mL, 78.13 pg/mL, 156.25 pg/mL, 312.50 pg/mL, 625 pg/mL, 1250 pg/mL, 2500 pg/mL, and 5000 pg/mL of each cytokine standard curve. Fitting standard curve for each cytokine, read the final concentration of each cytokine in each sample for statistical analysis.

### 2.5. Statistical Analysis

Gensini score exhibits a log-normal distribution after transformed as log(*X* + 2.5), and used as a dependent variable in multivariate linear regression model. Measurement data were described by mean ± standard deviation; after one-sample Kolmogorov-Smirnov normality test and variance homogeneity test, variables conforming to normal distribution and homogeneity of variance were tested by *T*-test, while variables with uneven variance were tested by correction *T*-test. The rest of the skewed distribution data was described with median, upper, and lower quartile. Counting data is tested by Chi-square. The concentration of cytokines in the two groups were compared with nonparametric Mann-Whitney *U* test. Spearman correlation analysis was used in two variables. All *P* values are derived from 2-tailed analyses; *P* < 0.05 have been considered to be of statistical significance. Analyses were performed with IBM SPSS statistics 26.

## 3. Results

### 3.1. Clinical Data

In this study, 101 patients were included at last, including 52 patients in the health control (HC) group and 49 patients in the CHD group, whose systolic blood pressure was higher than that in the control group (*P* < 0.05). In addition, compared to the CHD group, the HC group had lower triglyceride levels and higher HDL-c, which is consistent with current perceptions of CHD risk factors. In our study, there were no differences in T2DM distribution or GHbA1c levels between the two groups (*P* > 0.05), which can be used to analyze the difference of GLP-1R expression in macrophages and their subtypes in the two groups. The clinical and biochemical characteristics of the two groups in our research are as follows (Table 1).

### 3.2. Expression Differences of Total Macrophages, M1 Macrophages, and M2 Macrophages in CHD Group and HC Group: CD14^+^, CD14^+^CD80^+^, and CD14^+^CD206^+^

**Figure 1:**
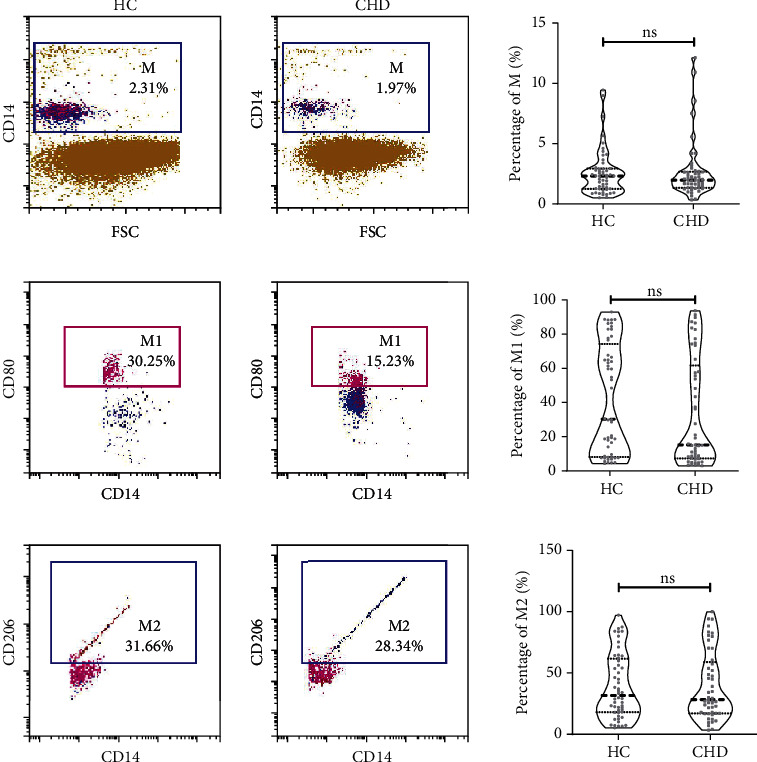
Expression difference of total macrophage, M1, and M2 macrophage between CHD group and HC group. Data are represented as mean ± SEM. ^∗^*P* < 0.05, ^∗∗^*P* < 0.01. M: macrophage, labeled by CD14+ molecule; M1: M1 macrophage, labeled by CD14+ and CD80+ molecule; M2: M2 macrophage, labeled by CD14+ and CD206+ molecule.

Total macrophages were labeled by CD14*^+^* antibody, M1 macrophages by CD14*^+^*CD80*^+^* double-positive antibody, and M2 macrophages by CD14*^+^*CD206*^+^* double-positive antibody. Previous studies have suggested that GLP-1R agonists can improve the risk of cardiovascular disease in diabetic patients, while in this study, there was no difference in the distribution of T2DM between the CHD group and the HC group, thus avoiding the bias caused by the difference in the distribution of diabetic patients between the two groups. In this study, the percentage of total, M1, and M2 macrophages showed no difference between the CHD group and the HC group (*P* > 0.05), indicating that the relative content of macrophages may not be the most critical factor for the development of CHD (Figure 1).

### 3.3. Expression Differences of GLP-1R on the Surface of Total, M1, and M2 Macrophages in CHD Group and HC Group: CD14^+^GLP-1R^+^, CD14^+^CD80^+^GLP-1R^+^, and CD14^+^CD206^+^GLP-1R^+^

**Figure 2:**
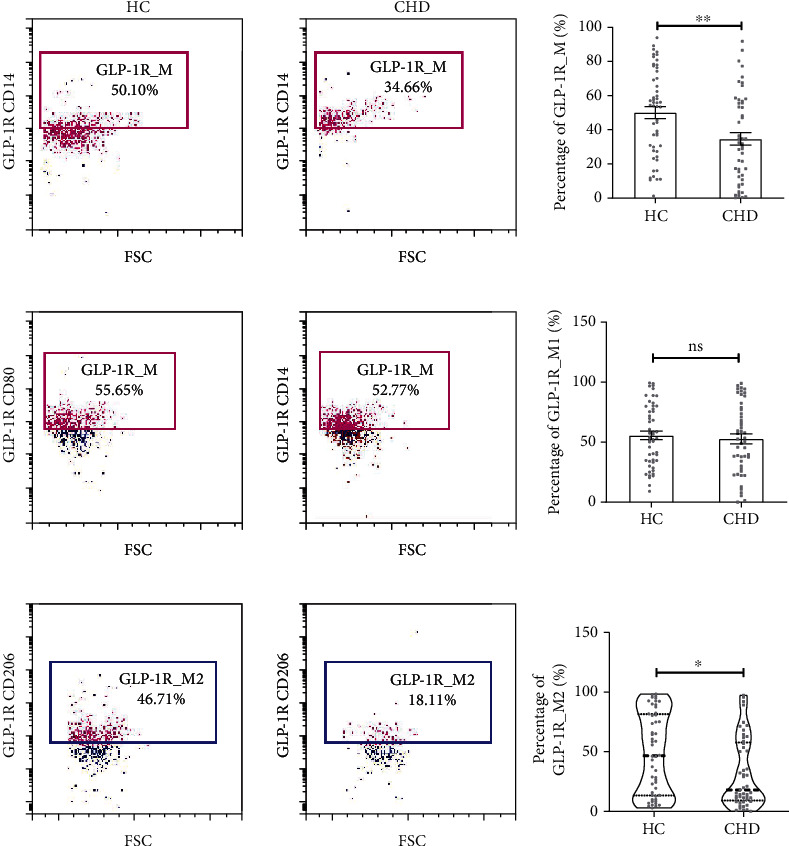
Expression difference of GLP-1R on the surface of total macrophage, M1, and M2 macrophage between two groups. Data are represented as mean ± SEM. ^∗^*P* < 0.05, ^∗∗^*P* < 0.01. GLP-1R_M: GLP-1R on the surface of macrophage, labeled by CD14+ and GLP-1R+ molecule; GLP-1R_M1: GLP-1R on the surface of M1 macrophage, labeled by CD14+, CD80+, and GLP-1R+ molecule; GLP-1R_M2: GLP-1R on the surface of M2 macrophage, labeled by CD14+, CD206+, and GLP-1R+molecule.

As mentioned earlier, no difference was found between the two groups in macrophages of different phenotypes, which does not seem to be synergistically demonstrated with the M1 and M2 macrophages effect that we commonly think of. However, we were surprised to find that GLP-1R expression on the surface of macrophages was different in both groups, although the percentage of different phenotypes of macrophages in the CHD group, and the control group was not statistically significant (*P* < 0.05), compared with the control group, the GLP-1R expression on M2 macrophages was higher in the CHD group, and this seems to indicate that macrophages with anti-inflammatory functions can express more cardiovascular beneficial GLP-1R. No statistically significant differential expression of anti-inflammatory phenotype was found in M1 macrophages (Figure 2).

### 3.4. Expression of IL-8, IL-1*β*, IL-6, IL-10, TNF, and IL-12P70 in the Two Groups

**Figure 3:**
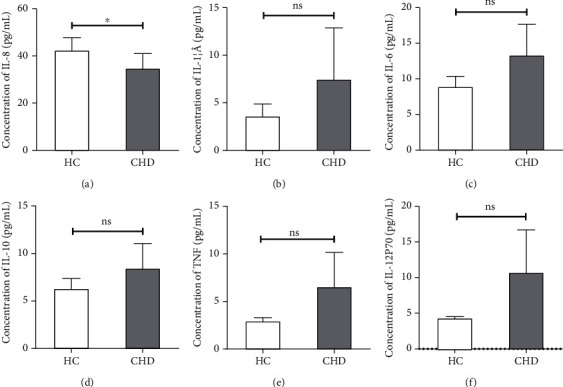
Plasma cytokine concentrations of IL-8, IL-1, IL-6, IL-10, TNF, and IL-12P70 in patients with coronary heart disease and health control. Data are represented as mean ± SEM. ^∗^*P* < 0.05, ^∗∗^*P* < 0.01.

There was a statistically significant difference in IL-8 concentrations between the two groups (*P* < 0.05), while none of the cytokines in IL-1*β*, IL-6, IL-10, TNF, and IL-12P70 were found to be different between the two groups in our study (Figure 3).

### 3.5. Correlation between Macrophages and Their Surface GLP-1R and Blood Lipids and Cytokines

**Figure 4:**
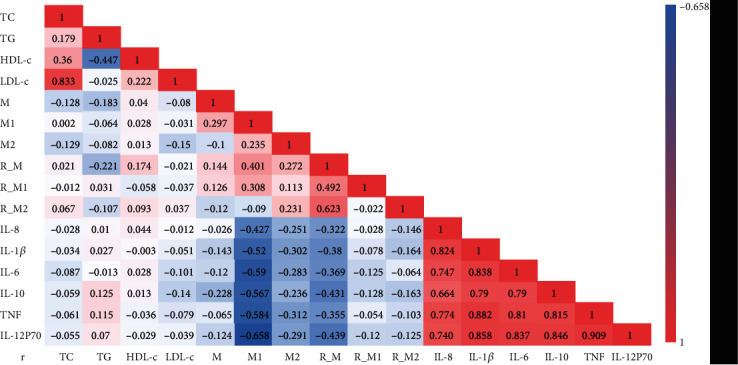
Correlation analysis between lipid markers, macrophage, GLP-1R, and cytokines in total 101 people. Spearman rank correlation was used. Value is correlation coefficient, bold black font represents *P* < 0.05, bold red font means *P* < 0.01. Colors represent different correlation coefficient as indicated by the color bar. R_M: GLP-1R_M; R_M1: GLP-1R_M1; R_M2: GLP-1R_M2.

We analyzed the correlation between macrophages and their surface GLP-1R and blood lipids and cytokines: (1) in all the studies included, GLP-1R expression on the surface of total macrophages was inversely proportional to TG (*r* = −0.221, *P* < 0.05), the percentage of M1 macrophages was inversely proportional to IL-10 (*r* = −0.228, *P* < 0.05); (2) in the HC group, the percentage of total macrophages was negatively correlated with TG (*r* = −0.306, *P* < 0.05), while the M2 macrophage GLP-1R expression increased with the decrease of IL-8 level (*r* = −0.275, *P* < 0.05); (3) in the CHD group, the percentage of M2 macrophages was inversely proportional to the percentage of total macrophages (*r* = −0.328, *P* < 0.05). However, the expression of GLP-1R in M1 macrophages was directly proportional to TG (*r* = 0.325, *P* < 0.05) (Figure4).

### 3.6. Effect of M1 Macrophage Percentage on Gensini Score

Log-transformed Gensini score was used as the dependent variable to adjust the potential confounders age, gender, smoking status (S.S.), drinking status (D.S.), HR, SBP, DBP, PP, TC, TG, HDL-C, LDL-C, GHbA1c, M, M1, M2 GLP-1R_M, GLP-1R_M1, GLP-1R_M2, IL-8, IL-1*β*, IL-6, IL-10, TNF, and IL-12p70. Multiple linear regression analysis was conducted, and the model equation was *Y* = 0.024∗Age + 0.497∗S.S.−0.454∗D.S.−0.003∗M1, and the equation was statistically significant (*P* < 0.05). The coefficient of determination R2 was 0.457, the entry condition was 0.1, and the exclusion condition was 0.15.

## 4. Discussion

The REWIND experiment [12] is a double-blind randomized placebo-controlled trial, which found that both middle-aged and elderly patients with T2DM who had previous cardiovascular diseases or had cardiovascular disease risk factors could benefit from the use of liraglutide. This suggests that the beneficial inhibitory effects of GLP-1R agonists on the occurrence and progression of cardiovascular diseases and the mechanism may not entirely dependent on the treatment of T2DM; therefore, our study included patients with CHD or not confirmed by coronary angiography and compared the distribution of macrophages and their surface GLP-1R without bias caused by the difference in T2DM distribution or GHbA1c level between the two groups. Data shows that the expression of GLP-1R on total and M2 macrophages was different between the CHD group and HC group (*P* < 0.05), and the expression of GLP-1R was higher in the HC group. The expression level of GLP-1R on the surface of M1 macrophages or the percentage of total, M1, and M2 macrophages showed no significant difference between the two groups. This suggests that: (1) in the chronic inflammatory process of CHD, changes in GLP-1R expression level are more sensitive than relative macrophage content; (2) the reduction of GLP-1R on beneficial anti-inflammatory type (M2) macrophage in CHD patients may explain that GLP-1R receptor agonists are independent of glycemic control effects, thus benefits for CHD.

DPP-4 inhibitors can inhibit the degradation of human native GLP-1 by DPP-4 enzyme and indirectly stimulate GLP-1R. Jawahar L. Mehta et al. [13] used PMA to block THP-1 macrophages and coincubate them with ox-LDL; inhibition of NLRP3 Toll-like receptor (TLR4) and IL-1 expression by DPP-4 inhibitors was observed, as well as upregulation of GLP-1R expression; while the blocking effect of PMA was relieved by GLP-1R agonists such as liraglutide, this study further demonstrates the inflammatory state and immune response of atherosclerosis. Our data shows that GLP-1R expression levels were indeed higher in total and M2 macrophages in the control group and not the percentage of the inflammatory cells itself, which suggests that GLP-1R may play a more central role than inflammatory cells themselves in the regulation of inflammatory nodes in atherosclerosis; in turn, it also supports the anti-inflammatory effect of GLP-1R in CHD.

Existing studies have shown that inflammatory response plays a vital role in the occurrence and development of CHD, T2DM, chronic obstructive pulmonary disease (COPD), chronic hepatitis, periodontitis [14], Alzheimer's disease (AD), etc. IL-6 [15, 16] has been shown to be negatively correlated with heart rate variability (HRV) in CHD women, elevated HRV is associated with parasympathetic excitation, the increased threshold of ventricular fibrillation is a protective factor, and this study reconfirms that IL-6 is a risk factor for CHD. Besides, IL-8, IL-10, and MCP-1, as a classic inflammatory cytokine, have been extensively studied in CHD [17–19]. Among the 6 cytokines involved in our study, IL-10 was the only anti-inflammatory cytokine, while the rest were proinflammatory cytokines. Nevertheless, our data showed that there was a statistically significant difference in IL-8 concentrations between the two groups (*P* < 0.05), while IL-1*β*, IL-6, TNF, and IL-12P70 revealed no significant difference.

Similar to the results of Karina Vargas-Sanchez's result, GLP-1R was not associated with any inflammatory markers [20]. Since the surface of natural killer T cells (NKT) also expresses GLP-1R, and this pathway can enhance the expression of anti-inflammatory cytokines such as IL-10 [21]; this may partly explain the higher IL-10 levels in Karina Vargas-Sanchez and our study in the disease group, although there was no statistically significant difference in IL-10 between the two groups in our study. Moreover, our sample is peripheral blood-derived cytokines, which can well reflect the downstream results of the aforementioned systematic regulation of human effects.

Other possibilities we speculate about for the above phenomenon are as follows: (1) since the diagnostic standard of CHD is 50% of the stenosis degree of one or more major coronary arteries as the limit, the subjects who did not reach the stenosis degree but still had slight plaque formation were included in the HC group, leading to the fact that the HC group was not completely free of plaque formation, but more than that, if a statistically significant difference was found between the CHD group and the population with stenosis between 0 and 50 percent, it would be easier to identify patients without stenosis at all; (2) compared with acute inflammation caused by severe trauma or pathogen infection, CHD is a mild chronic inflammation of the circulatory system [19], with low baseline concentration of inflammatory or anti-inflammatory cytokine and no significant change in a short term; it is possible that a large sample size of a multicenter, large-scale population would reveal statistical differences [17]; (3) mild inflammation is common in a variety of chronic diseases, and the exclusion criteria of this study is limited to acute inflammation, infective inflammation, and patients with autoimmune diseases or take immunosuppressant, etc., other than cardiovascular system of chronic inflammatory diseases [22] such as mild liver dysfunction or mild chronic gastritis patients has not been ruled out strictly; what is more, too strict rule out conditions limit the extrapolation of the results of the study and against the scenario of the real world; (4) IL-1, IL-12, and TNF-*α* can be used as the characteristic molecule of M1 macrophages, while IL-10 and IL-4 can be used as the characteristic molecule of M2 macrophages [23]. As mentioned in this study, there was no difference in the percentage of M1 macrophages or M2 macrophages between the HC group and the CHD group; this may explain why IL-12, IL-4, IL-1, and IL-10 were not different between the two groups.

Atherosclerosis is closely related to lipid metabolism and inflammatory reaction [24, 25]. In the 101 people included in this study, correlation analysis shows that the GLP-1R expression level of total macrophages was inversely proportional to TG; TG is a known and recognized cardiovascular risk factor [26], indicating that GLP-1R negatively regulates TG in both patients with CHD and CS population. In the HC group, the expression level of GLP-1R M2 was negatively correlated with IL-8, as a chemokine of neutrophils; IL-8 promotes the migration of neutrophils to the inflammatory site and blocks cholesterol efflux by inhibiting the expression of ABCA1 [27], thus promoting the inflammatory response. Laura J. den Hartigh [28] stimulated human monocytes with TG and lipoprotein lipase, the degradation product of VLDL, then observed that monocytes initiate adhesion to endothelial cells, and detected the up-regulation of IL-8 expression, thus supporting our results. Combined with flow cytometry detection in this study, it was found that the expression level of M2 macrophages in the HC group was higher than that in the CHD group; we speculated that, compared with the CHD group, the enhanced protective factors represented by the up-regulated GLP-1R expression of M2 macrophages in the HC group were the main reasons.

Though in the CHD group, the expression level of GLP-1R_M1 was positively correlated with TG, and we have observed that GLP-1R on the cell membrane of total, M1, and M2 macrophages was lower than that of the HC group. We thus speculated that the high expression of GLP-1R was related to the increase in the number of M1 macrophages, or, as a negative feedback regulatory compensation mechanism of the body, M1 macrophages could be highly expressed in GLP-1R. Although this study did not show a difference in the percentage of M1 macrophages between the two groups, clinical studies with a larger sample size may show a difference to explain this phenomenon, and the specific mechanism and causal relationship need to be further studied. Luo et al. [29] inhibited the ROS, NLRP3, and Caspase-1 of HUVECs by using GLP-1R agonist Dulaglutide and also inhibited the maturation of IL-1 and IL-18. Therefore, GLP-1R may mediate the polarization of macrophages and release different inflammatory small molecules through NLRP3/Caspase1 mechanism, so as to regulate the development of AS.

The massive immune system composed of immune cells, the immune organs, and the inflammatory response participates in physiological and pathological conditions of the body all the time; the role and mechanism of the newly discovered macrophage-subtype T cell subtype in atherosclerosis need to be further studied. Like most clinical studies, CHD group and the HC group are according to the diagnostic gold standard of coronary angiography and intravascular ultrasound to distinguish, but the technology itself, provides only stenosis site and degree of structural information, such as functional index score blood flow reserve (FFR) has not been collected and assist for the illness subgroup analysis. Last but not least, it is hoped to find disease biomarkers of CHD [30], such as C-reactive protein (CRP), IL-6, serum amyloid A (SAA), CD40/CD40 ligand (CD40L), and heat shock protein 60 (Hsp60) [31] and 70 (Hsp70), but their specificity needs to be improved; therefore, more sensitive and specific inflammatory disease biomarker still need to be studied.

## 5. Conclusion

GLP-1R agonist is independent of the hypoglycemic effect of T2DM and has protective effect on cardiovascular system. GLP-1R may regulate the polarization of macrophages toward M2, thus playing a protective role in the progression of coronary atherosclerosis.

## Figures and Tables

**Table 1:** Clinical and biochemical characteristics in HC and CHD groups.

	HC group	CHD group	*P* value
	(*n* = 52)	(*n* = 49)	
Age (years)	56.71 ± 10.15	63.59 ± 11.22	0.002
Gender (male, female)	27/25	32/17	0.173
Smoking status (yes, no)	14/38	22/27	0.059
Drinking status (yes, no)	6/46	8/41	0.486
HR (/bmp)	75 (66.25~83.5)	75 (64.5~83.5)	0.534
SBP (mmHg)	127.5 (120~141)	139 (126.5~150.5)	0.015
DBP (mmHg)	78.5 (70.25~87.5)	80 (72~88)	0.809
PP (mmHg)	51.5 (43.5~60)	57 (46~68.5)	0.087
TC (mmol/L)	4.05 (3.54~5.04)	4.21 (3.36~4.98)	0.943
TG (mmol/L)	1.33 (0.83~1.11)	1.81 (1.31~2.84)	0.009
HDL-c (mmol/L)	0.98 (0.86~1.11)	0.90 (0.73~1.14)	0.043
LDL-c (mmol/L)	2.22 (1.82~3.06)	2.01 (1.54~2.94)	0.213
GHbA1c (%)	5.5 (5.3~5.9)	5.8 (5.3~6.75)	0.126
T2DM (yes, no)	5/47	10/39	0.127

HC: health control; CHD: coronary heart disease; HR: heart rate; SBP: systolic blood pressure; DBP: diastolic blood pressure; PP: pulse pressure; TC: cholesterol; TG: triglyceride; HDL-c: high-density lipoprotein cholesterol; LDL-c: low-density lipoprotein cholesterol; GHbA1c: glycosylated hemoglobin A1c; T2DM: type 2 diabetes mellitus.

## Data Availability

Supporting data of the findings in our study are available from the corresponding author upon request.
